# Cariporide Attenuates Doxorubicin-Induced Cardiotoxicity in Rats by Inhibiting Oxidative Stress, Inflammation and Apoptosis Partly Through Regulation of Akt/GSK-3β and Sirt1 Signaling Pathway

**DOI:** 10.3389/fphar.2022.850053

**Published:** 2022-06-07

**Authors:** Wenli Liao, Zhiwei Rao, Lingling Wu, Yong Chen, Cairong Li

**Affiliations:** ^1^ National Demonstration Center for Experimental General Medicine Education, Xianning Medical College, Hubei University of Science and Technology, Xianning, China; ^2^ Central Hospital of Xianning, The First Affiliate Hospital of Hubei University of Science and Technology, Xianning, China

**Keywords:** cariporide, doxorubicin, cardiotoxicity, apoptosis, SIRT1, Na+/H+ exchanger isoform 1

## Abstract

**Background:** Doxorubicin (DOX) is a potent chemotherapeutic agent with limited usage due to its cumulative cardiotoxicity. The Na^+^/H^+^ exchanger isoform 1 (NHE1) is a known regulator of oxidative stress, inflammation, and apoptosis. The present study was designed to investigate the possible protective effect of cariporide (CAR), a selective inhibitor of NHE1, against DOX-induced cardiotoxicity in rats.

**Methods:** Male Sprague-Dawley rats were intraperitoneally injected with DOX to induce cardiac toxicity and CAR was given orally for treatment. The injured H9c2 cell model was established by incubation with DOX *in vitro*. Echocardiography, as well as morphological and ultra-structural examination were performed to evaluate cardiac function and histopathological changes. The biochemical parameters were determined according to the manufacturer’s guideline of kits. ROS were assessed by using an immunofluorescence assay. The serum levels and mRNA expressions of inflammatory cytokines were measured by using ELISA or qRT-PCR. Cardiac cell apoptosis and H9c2 cell viability were tested by TUNEL or MTT method respectively. The protein expressions of Cleaved-Caspase-3, Bcl-2, Bax, Akt, GSK-3β, and Sirt1 were detected by western blot.

**Results:** Treatment with CAR protected against DOX-induced body weight changes, impairment of heart function, leakage of cardiac enzymes, and heart histopathological damage. In addition, CAR significantly attenuated oxidative stress and inhibited the levels and mRNA expressions of inflammatory cytokines (TNF-α, IL-6, IL-18, and IL-1β), which were increased by DOX treatment. Moreover, CAR significantly suppressed myocardial apoptosis and Cleaved-Caspase-3 protein expression induced by DOX, which was in agreement with the increased Bcl-2/Bax ratio. Also, DOX suppressed phosphorylation of Akt and GSK-3β, which was significantly reversed by administration of CAR. Furthermore, CAR treatment prevented DOX-induced down-regulation of Sirt1 at the protein level *in vitro* and *in vivo*. Finally, Sirt1 inhibitor reversed the protective effects of CAR, as evidenced by reduced cell viability and Sirt1 protein expression *in vitro*.

**Conclusion:** Taken together, we provide evidence for the first time in the current study that CAR exerts potent protective effects against DOX-induced cardiotoxicity in rats. This cardio-protective effect is attributed to suppressing oxidative stress, inflammation, and apoptosis, at least in part, through regulation of Akt/GSK-3β and Sirt1 signaling pathway, which has not been reported to date.

## Introduction

As an anthracycline drug, doxorubicin (DOX) is one of the most extensively used chemotherapeutic agents for treatment of various cancers including leukemia, lymphoma, breast cancer, and other solid tumors (Rivankar, 2014). Unfortunately, despite its remarkable anticancer activity, the clinical application of DOX is markedly limited by its serious cardiotoxicity, which is characterized by electrocardiographic changes, cardiac arrhythmia, and irreversible degenerative cardiomyopathy (Smith et al., 2010). DOX-induced cardiotoxicity can occur immediately, within months, or even years after DOX treatment (Alkreathy et al., 2010). The exact molecular mechanisms responsible for DOX-induced cardiotoxicity are still not fully understood, and few drugs have been tested clinically to alleviate DOX-induced cardiotoxicity. Numerous studies have implicated that reactive oxygen species (ROS) generation, mitochondrial dysfunction, inflammation, apoptosis and various signaling pathways were convincingly shown to be crucial in DOX-induced cardiotoxicity (Mukhopadhyay et al., 2007; Meeran et al., 2019; Chen et al., 2020).

Sirtuin 1 (Sirt1), an NAD^+^-dependent histone deacetylase, plays important roles in multiple biological processes including longevity, stress response, and cell survival. It has been well established that Sirt1 was involved in redox regulation, cell apoptosis, as well as inflammation (Hwang et al., 2013), and the protein level of Sirt1 increased in response to DOX injection (Zhang et al., 2011). Some evidence supports that PI3K-Akt-GSK3β signaling pathway is necessary for endoplasmic reticulum stress-induced Sirt1 activation (Koga et al., 2015), and the protective role of PI3K/Akt signaling pathway is found in DOX-induced cardiac dysfunction (Wen et al., 2019). The activation of PI3K/Akt signaling pathway can suppress DOX-induced cardiomyocyte apoptosis. In particular, GSK-3β is a downstream effector of PI3K/Akt signaling pathway and can lead to the mitochondrial permeability transition pore opening and subsequently apoptosis (Townsend et al., 2007). As DOX still remains a mainstay of many chemotherapeutic regimens, further investigation of its cardiotoxicity and how to prevent it is warranted.

Cariporide (CAR) is a selective Na+/H+ exchanger isoform 1 (NHE1) inhibitor, which can significantly improve DOX sensitivity in a xenograft model, specifically enhancing tumor growth inhibition and reducing tumor volume (Chen et al., 2019). CAR also reverses burn-induced intracellular Na + accumulation and cell apoptosis involved in PI3K/Akt and p38 MAPK pathways (Fang et al., 2020). Inhibition of NHE1 exerts potent cardioprotective effects against ischemia/reperfusion-induced heart injury through activation of Akt/GSK-3β survival pathway (Jung et al., 2010). Additionally, gene inactivation of NHE1 attenuates transient focal cerebral ischemia induced-apoptosis and mitochondrial injury (Wang et al., 2008). NHE1 inhibition also ameliorates peripheral diabetic nephropathy, as well as alleviates atherosclerotic lesion growth and promotes plaque stability by inhibiting the inflammatory reaction (Li et al., 2014). Moreover, inhibition of NHE1 by its inhibitor amiloride significantly enhances the intracellular accumulation of DOX in DOX-resistant human colon cancer cells and thereby increases their treatment (Miraglia et al., 2005). A recent study reported that citronellal could ameliorate DOX-induced cardiotoxicity by inhibiting the NHE1-mediated oxidative stress, apoptosis in rats (Liu X. et al., 2021). Considering the effects of NHE1 in oxidative stress, inflammation, apoptosis and sensitivity of DOX, we hypothesized that its selective inhibitor CAR not only enhanced the anticancer effect of DOX, but also might have the protective effect against DOX-induced cardiotoxicity.

Therefore, the present study was undertaken to investigate the protective effects of CAR against DOX-induced cardiotoxicity in rats, and to elucidate the underlying mechanisms of its cardioprotective effects. Our findings demonstrated that CAR could alleviate DOX-induced cardiotoxicity via suppression of oxidative stress, inflammation and apoptosis, which was at least partially through regulation of Akt/GSK-3β and Sirt1 signaling pathway. The potent protective effects of CAR against DOX-induced cardiotoxicity in rats have not been reported to date, and it is the first time to report Sirt1 signaling pathway is involved in the cardio-protective effect of CAR. These results reveal a novel role of CAR in DOX-induced cardiotoxicity and suggest that NHE1 may be a therapeutic target for lessening DOX-induced cardiac damage.

## Materials and Methods

### Materials

CAR was purchased from Santa Cruz Biotechnology (Santa Cruz, CA, United States). DOX and nicotinamide were purchased from Aladdin Technology (Shanghai, China). Anti-Bcl-2, anti-Bax, anti-Akt, anti-GSK-3β, anti-phospho-Akt, anti-phospho-GSK-3β, anti-Sirt1, and anti-GAPDH were purchased from Santa Cruz Biotechnology (Santa Cruz, CA, United States). The test kits of creatine kinase-MB fraction (CK-MB), lactate dehydrogenase (LDH), malondialdehyde (MDA), superoxide dismutase (SOD), glutathione peroxidase (GSH-Px) and catalase (CAT) were purchased from Nanjing Jiancheng Biotechnology Institute (Nanjing, China). The CCK-8 kit was purchased from Wuhan Saiweier Biotechnology Co., Ltd (Wuhan, China). The test kit of cTnT was purchased from Milliplex Company (Darmstadt, Germany). The ELISA test kits of tumor necrosis factor-alpha (TNF-α), interleukin-6 (IL-6), interleukin-18 (IL-18) and interleukin-1β (IL-1β) were purchased from Dakewe Biotec Company (Beijing, China). The *In Situ* Cell Death Detection Kit was purchased from Roche Company (Mannheim, Germany). TRIzol reagent was purchased from Invitrogen Corporation (Carlsbad, CA, United States). All other chemicals were purchased in the highest grade available from Sigma-Aldrich Corporation (St. Louis, MO, United States).

### Animals and Experimental Design

Thirty-two adult male Sprague-Dawley rats weighing 280–310 g were obtained from the Experimental Animal Research Center of Hubei Province (Certificate No. SCXK [E] 2015–0018, Wuhan, China). All animal procedures and experiments described in this study were approved by the Review Committee for the Use of Human or Animal Subjects of Hubei University of Science and Technology. The animals were housed under controlled environmental conditions of humidity (40%–50%) and temperature (25 ± 2°C) with natural light and dark cycles (12 h∶ 12 h) and were allowed free access to food and water.

Rats were randomly divided into four groups (8 per group): Control group (CON), DOX group (DOX), DOX with CAR treatment group (DOX + CAR), and CAR group (CAR). Rats in CON group were received standard laboratory diet and drinking water. Rats in DOX group were intraperitoneally (i.p.) injected with DOX (2.5 mg/kg, every other day) over a period of 12 days for a cumulative dose of 15 mg/kg as described previously (Siveski-Iliskovic et al., 1994). Rats in DOX + CAR group were injected with DOX (i.p.) at a dose of 2.5 mg/kg every other day for a cumulative dose of 15 mg/kg and simultaneously treated with CAR (1 mg kg^−1^ day^−1^) once a day over a period of 12 days. Rats in CAR group were treated with CAR (1 mg kg^−1^ day^−1^) once a day over a period of 12 days.

### Detection of Myocardial Injury Markers

The levels of myocardial enzymes CK-MB, and LDH activities in the serum, which were considered as the pivotal diagnostic indicators of myocardial injury, were tested following the manufacturer’s protocols (Nanjing Jiancheng Biotechnology Institute, China). The level of cTnT was measured according to the manufacturer’s guideline (Milliplex Company, Darmstadt, Germany).

### Assessment of Left Ventricular Function

At the end of the experiment, transthoracic echocardiography was performed in all groups under isoflurane (1%–3%) anesthesia using an echocardiography system (Vevo 2100, VisualSonics, Canada). The echocardiography parameters were as follows: left ventricular end-diastolic diameter (LVEDD) and left ventricular end-systolic diameter (LVESD). To assess left ventricular systolic function, the ejection fraction (EF) and fractional shortening (FS) were also calculated.

### Morphological and Ultra-Structural Examination

The left ventricles of the heart samples were removed and fixed by immersion in 10% formalin. Subsequently, parts of the left ventricles were embedded in paraffin wax, cut into 3-μm-thick sections, stained with hematoxylin-eosin (HE) staining or Masson’s staining respectively, and examined under a light microscope (CKX41, 170 Olympus, Tokyo, Japan) at total magnifications of ×400 by a pathologist blinded to this study. For ultra-structural examination, the samples were immersed with 2.5% glutaraldehyde for 2 h and were fixed in 1% osmic acid for 3 h. After embedding in paraffin, ultra-thin sections (60–80 nm) were stained with 3% uranyl acetate and lead citrate, and were then examined by transmission electron microscope (TEM, HT7700 120 kv, HITACHI, Japan). The qualitative analysis of histopathological changes was performed as none (-) to severe (+++) according to the degree of inflammation, myocardial disorganization, and myofibrillar loss, which are listed in Table 1. The scoring system was as follows (-) no damage, (+) mild damage, (++) moderate damage, and (+++) severe damage.

**TABLE 1:** Effect of CAR on morphological changes as assessed by histopathological examination of hearts from the DOX-treated rats.

Groups	n	Inflammation	Myocardial Disorganization	Interstitial Fibrosis
CON	12	(-)	(-)	(-)
DOX	9	(+) to (++)	(++) to (+++)	(+) to (++)
DOX + CAR	13	(-) to (+)	(+) to (++)	(-) to (+)
CAR	12	(-)	(-)	(-)

(–): none; (+): mild; (++): moderate; (+++): severe.

### Assessment of Biochemical Parameters

The content of MDA and the activities of antioxidant enzymes including SOD, GSH-Px, and CAT in heart homogenates were measured according to the manufacturer’s instructions (Nanjing Jiancheng Biotechnology Institute, China). The levels of TNF-α, IL-6, IL-18, and IL-1β in serum were determined with ELISA test kits from Dakewe Biotec Company (Beijing, China) following the manufacturer’s protocols.

### Cell Culture and Detection of Cellular ROS

The H9c2 cell line was purchased from the China Center for Type Culture Collection (CCTCC, China), and cultured in Dulbecco’s modified Eagle’s medium with fetal bovine serum (10%), streptomycin (1%), and penicillin. The culture conditions contained a humidified atmosphere (95% air and 5% CO_2_ at 37°C). The H9c2 cells were incubated with DOX (1 μmol/L) for 72 h with or without CAR(5 μmol/L) and Sirt1 inhibitor (nicotinamide, 10 μmol/L). The cell viability was detected by a CCK-8 kit. The ROS-level was measured by dihydroethidium (DHE, Beyotime Biotechnology, China), an indicative fluorescence probe, which was used to detect intracellular superoxide anions.

### Quantitative Real-Time PCR

Total RNAs were extracted from cardiac tissues by using the TRIzol reagent (TaKaRa, Japan) according to the manufacturer’s instructions. cDNA synthesis was performed with HiScriptIIQ RT SuperMix (Vazyme, China) according to the manufacturer’s instructions. Quantitative RT-PCR was performed with ChamQ SYBR qPCR Master Mix (Vazyme, China) according to the protocol. GAPDH was used as the reference gene.

### Terminal Deoxynucleotidyl Transferase-Mediated dUTP Nick End-Labelling Assay

Cardiac apoptotic cells were tested by TUNEL staining which was often used for detecting DNA fragmentation. The TUNEL assay was performed according to the manufacturer’s protocol and instructions provided in the *In Situ* Cell Death Detection Kit supplied by Roche Company (Mannheim, Germany). The results were examined and the apoptotic index was calculated under a light microscope (CKX41, 170 Olympus, Tokyo, Japan) at a total magnification of ×400 by a pathologist blinded to this study.

### Western Blot Analysis

Equal amounts of protein from hippocampal homogenates were separated by 10% SDS-PAGE gels and then transferred to PVDF membranes. The membranes were blocked for 1 h at room temperature in 5% non fat milk. After being incubated overnight at 4°C with the appropriate primary antibodies including Bcl-2, Bax, phospho-Akt, Akt, phospho-GSK-3β, GSK-3β, Sirt1, and GAPDH (Santa Cruz, CA, United States), the membranes were washed 15 min with TBST three times and then incubated with the secondary antibody for 1 h at room temperature. The blots were then imaged using ECL assay kits (Dalian Meilun Biotech Co., Ltd, China). The band intensities were quantified using NIH ImageJ 1.50 software and normalized to the quantity of GAPDH in each sample lane. All assays were performed at least three times.

### Statistics

The values are expressed as means ± SEM. Data were analyzed One-way ANOVA followed by post hoc Tukey’s test by employing GraphPad Prism Version 5.0. *p* values of 0.05 or less were considered to be statistically significant.

## Results

### CAR Reinstated Body Weight and Myocardial Marker Enzymes in DOX-Induced Cardiotoxicity in Rats

**FIGURE 1:**
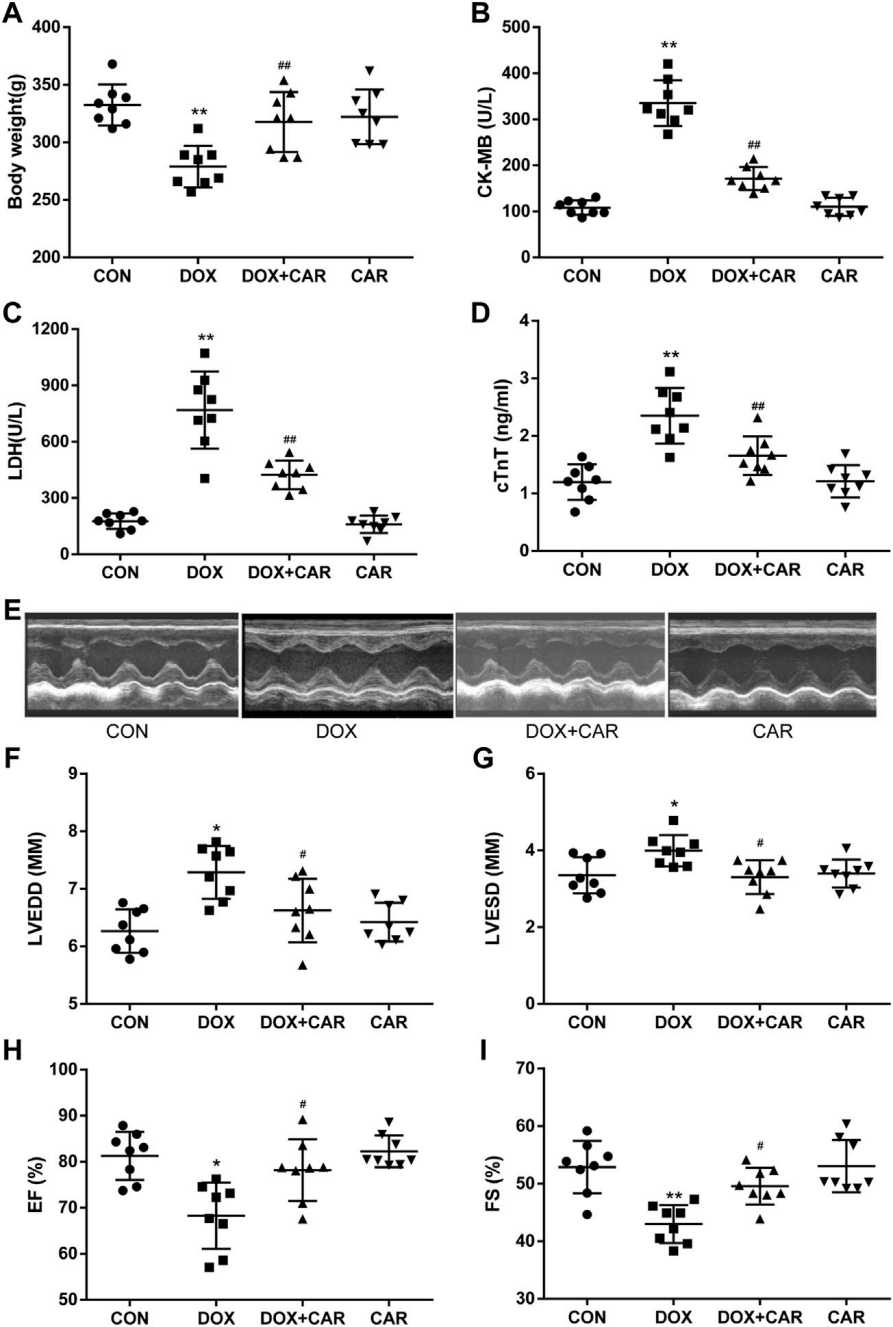
CAR reinstated body weight, myocardial marker enzymes and left ventricular dysfunction in DOX-induced cardiotoxicity in rats. **(A)** body weight, **(B)** LDH, **(C)** CK-MB, **(D)** cTnT, **(E)** Representative pictures of heart function, **(F)** LVEDD, **(G)** LVESD, **(H)** EF, and **(I)** FS. Quantitative data are means ± SEM (n = 8). One-way ANOVA followed by post hoc Tukey’s test. *p < 0.05, **p < 0.01 vs. CON; #*p* < 0.05, ##*p* < 0.01 vs. DOX.

As compared to CON group, the body weight dramatically decreased in DOX group (*p* < 0.01 vs. CON, Figure 1A). Administration of CAR resulted in a significant prevention of DOX-induced decrease in body weight (*p* < 0.01 vs. DOX, Figure 1A). To further confirm the protective effects of CAR against DOX-induced cardiotoxicity, we tested the serum levels of cardiac enzymes (CK-MB, LDH, and cTnT), which represented the biochemical markers of myocardial injury. As shown in Figures 1B–D, treatment with DOX caused significantly elevated serum levels of CK-MB, LDH, and cTnT as compared to CON group (*p* < 0.01 vs. CON for all). Administration of CAR (10 mg kg^−1^ day^−1^) caused a reversal of DOX-induced increase in serum cardiac enzymes (*p* < 0.01 vs. DOX for all). Moreover, no significant changes in body weight and serum cardiac enzymes were observed in CAR group as compared to CON group, demonstrating that the dose of CAR used in this study (10 mg kg^−1^ day^−1^) did not affect body weight and myocardial marker enzymes of the rats.

**FIGURE 2:**
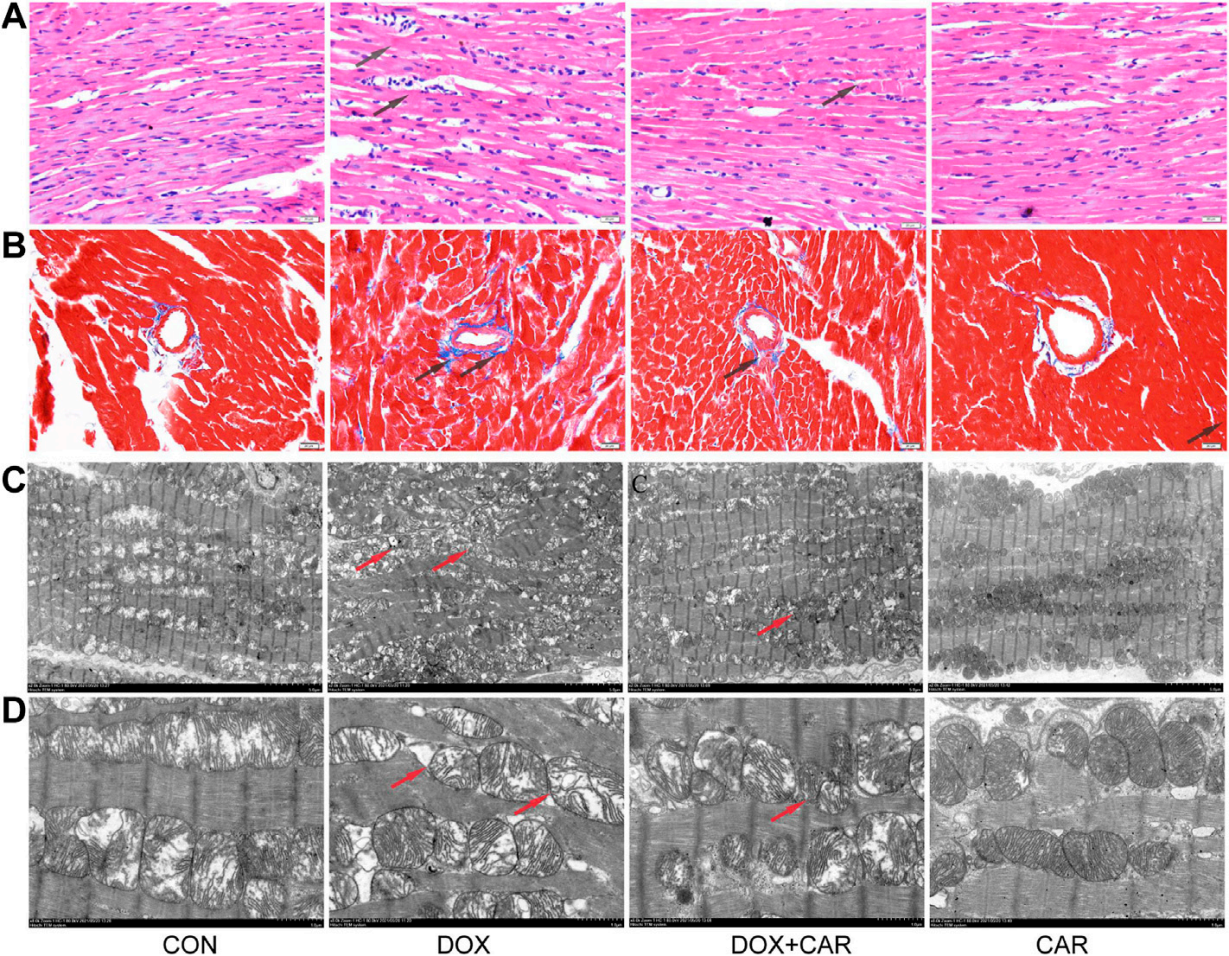
CAR attenuated the pathological alterations in myocardial tissues of DOX-treated hearts. Representative pictures of myocardial tissue sections stained with H&E and Masson (magnification = ×400) **(A)** H&E staining, **(B)** Masson; n = 3, Bar = 20 μm. Representative images of myocardial tissue sections tested by TEM **(C)**, the magnification is 1.5 K times (Scale = 5 μm). Representative images of myocardial tissue sections tested by TEM **(D)**, the magnification is 5.0 K times (Scale = 2 μm).

### CAR Prevented DOX-Induced Left Ventricular Dysfunction

Next, we conducted an echocardiography analysis of left ventricular function. Representative pictures of heart function were shown in Figure 1E. Administration of DOX resulted in a significant increase of LVEDD and LVESD (*p* < 0.05, *p* < 0.05 vs. CON), both of which were preserved by CAR treatment (*p* < 0.05, *p* < 0.05 vs. DOX, Figures 1F,G). Furthermore, FS and EF, the index of left ventricular systolic function, decreased dramatically in DOX group (*p* < 0.05, *p* < 0.01 vs. CON). CAR treatment significantly ameliorated the reduction of FS and EF caused by DOX (*p* < 0.05, *p* < 0.05 vs. DOX, Figures 1H,I). In addition, CAR alone had no effect on left ventricular function. These data suggest that CAR can ameliorate the impairment of heart function induced by DOX.

### Histopathological and Ultra-structural Analysis by HE Staining, Masson’s Staining, and TEM

**FIGURE 3:**
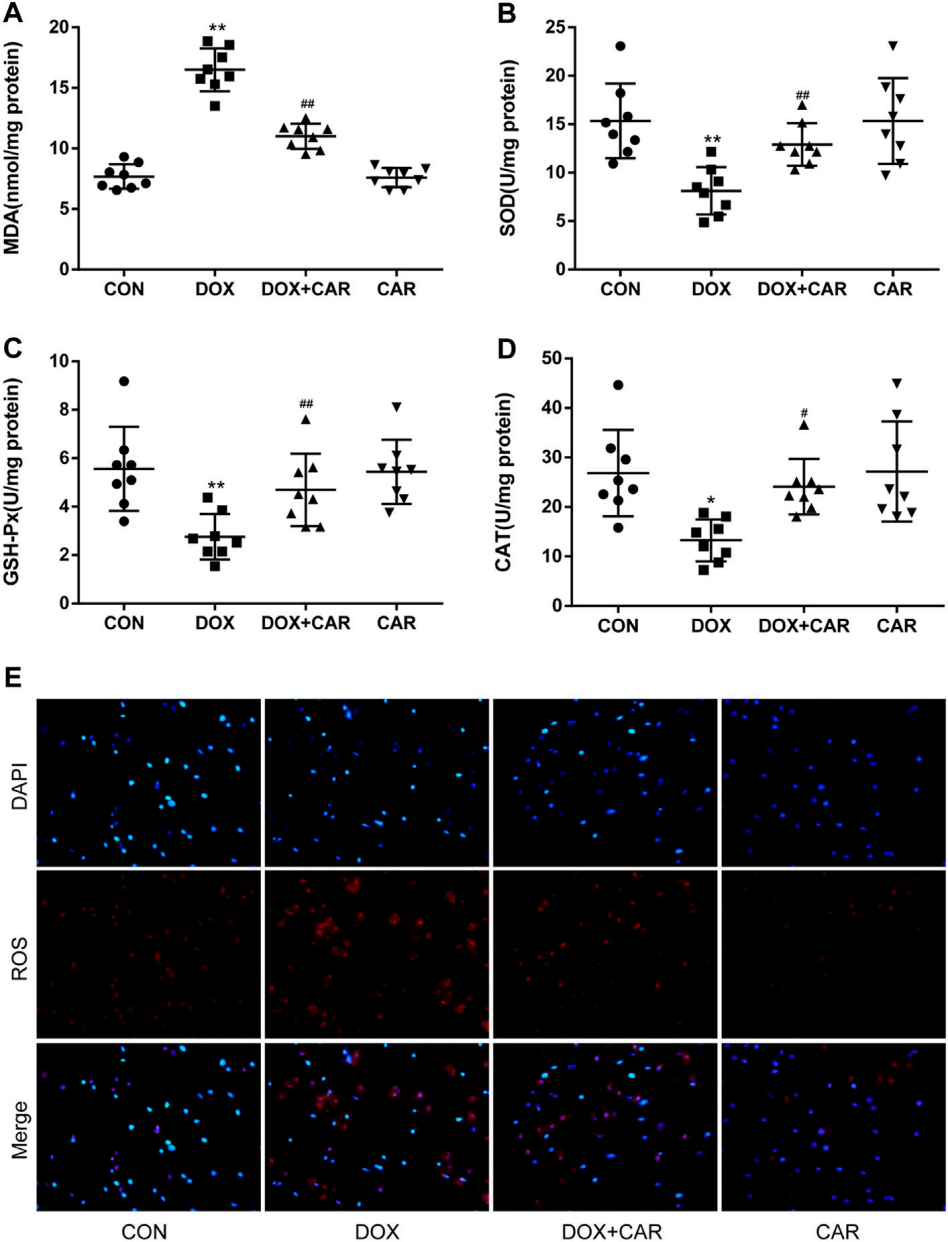
CAR alleviated oxidative stress in myocardial tissues in rat and H9c2 cells treated with DOX. Quantitative analysis **(A)** MDA, **(B)** SOD, **(C)** GSH-Px, and **(D)** CAT. Representative images of cells stained with DAPI and DHE **(E)**, Scale = 20 μm. Quantitative data are means ± SEM (n = 8). One-way ANOVA followed by post hoc Tukey’s test. *p < 0.05, **p < 0.01 vs. CON; #*p* < 0.05, ##*p* < 0.01 vs. DOX.

To assess cardiac morphological alterations, sections of rat heart tissue were stained with HE or Masson’s staining, and examined by light microscopy. As shown in Figure 3, DOX-induced cardiotoxicity was characterized by mild focal inflammation, myofibrillar loss, swelling, and fibrosis. Our results revealed that CAR treatment significantly ameliorated DOX-induced lesions on myocardial morphology (Figure 3A), and mitigated the cardiac fibrosis (Figure 3B). TEM performed for the myocardium showed that treatment with DOX caused marked ultra-structural aberration leading to myofibrillar disintegration, damage of Z-band and M-band, and irregular mitochondria (Figures 3C,D). The qualitative analysis of histopathological changes were shown in Table 1. Treatment with CAR markedly improved the irregular and disintegrated sarcomere, restored the Z-band and M-band, and reduced the damaged mitochondria.

**FIGURE 4:**
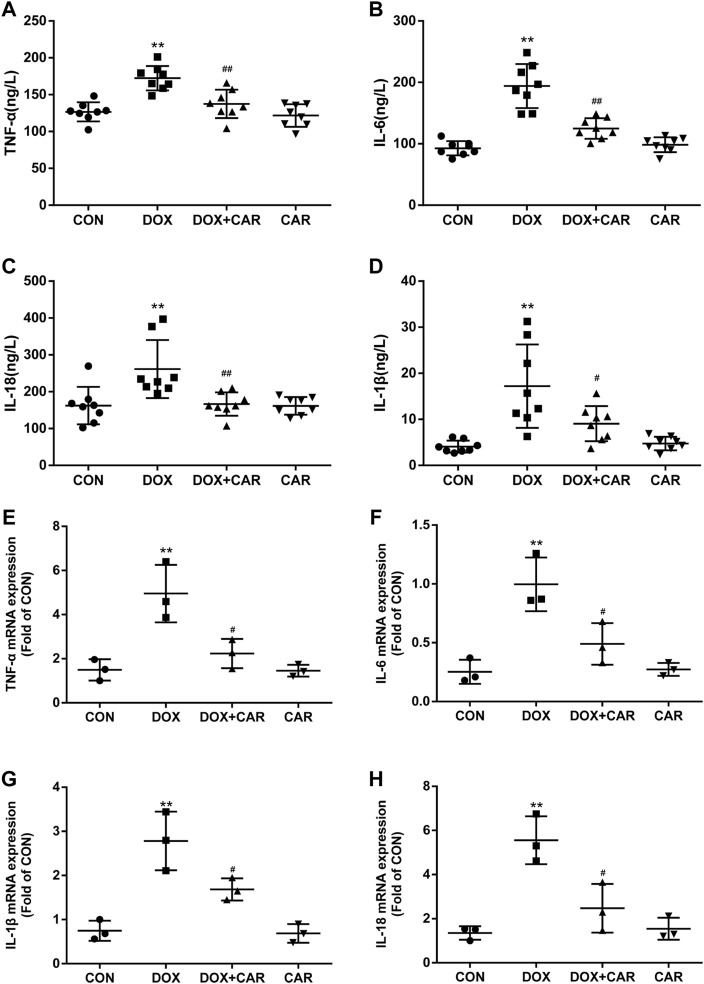
CAR alleviated the serum levels and mRNA expressions of inflammatory cytokines in DOX-treated rats. Quantitative analysis of serum levels **(A)** TNF-α, **(B)** IL-6, **(C)** IL-18 and **(D)** IL-1β; Quantitative data are means ± SEM (n = 8). Quantitative analysis of mRNA expressions **(E)** TNF-α, **(F)** IL-6, (G) IL-18 and **(H)** IL-1β. Quantitative data are means ± SEM (n = 3). One-way ANOVA followed by post hoc Tukey’s test. **p < 0.01 vs. CON; #*p* < 0.05, ##*p* < 0.01 vs. DOX.

### CAR Alleviated Oxidative Stress Induced by DOX

It is well established that oxidative stress plays a critical role in DOX-induced cardiotoxicity. Therefore we investigated the effects of CAR on markers of oxidative stress, including MDA and the antioxidant enzymes (SOD, GSH-Px, and CAT). As expected, DOX administration resulted in a significant increased level of MDA and a significant decreased activities of SOD, GSH-Px, and CAT as compared to CON group (*p* < 0.01, *p* < 0.01, *p* < 0.01, *p* < 0.05 vs. CON). However, treatment with CAR significantly prevented DOX-induced increased level of MDA and decreased activities of SOD, GSH-Px, and CAT (*p* < 0.01, *p* < 0.01, *p* < 0.01, *p* < 0.05 vs. DOX, Figures 3A–D). Administration of CAR alone had no significant effect on the level of MDA or the activities of SOD, GSH-Px, and CAT as compared to CON group.

DOX-induced ROS level *in vitro* was also detected by using DHE as a fluorescent probe. The results showed that H9c2 cells treated with DOX alone showed greater red fluorescence, indicating that DOX treatment led a clear increase in intracellular ROS levels. On the other hand, the red fluorescence in the cells treated with the combination of DOX and CAR exhibited largely reduced brightness when compared with those cells treated with DOX only, demonstrating that CAR could prevent intracellular ROS production (Figure 3E). These results suggest that CAR may protect against DOX-induced cardiotoxicity at least partially through suppression of oxidative stress.

### Effect of CAR on Inflammation Following DOX-Induced Cardiotoxicity

Since several inflammatory cytokines are associated with the pathological injury caused by DOX-induced cardiotoxicity, the serum levels and mRNA expressions of TNF-α, IL-6, IL-18, and IL-1β in heart tissues were then tested. As expected, the serum levels and cardiac mRNA expressions of TNF-α, IL-6, IL-18, and IL-1β were dramatically elevated in DOX group (*p* < 0.01 vs. CON for all, Figures A, B, C, and D indicated serum levels of TNF-α, IL-6, IL-18, and IL-1β; Fig. E, F, G and H indicated mRNA of TNF-α, IL-6, IL-18, and IL-1β). On the other hand, treatment with CAR protected against DOX-induced increase of serum levels and cardiac mRNA expressions of TNF-α, IL-6, IL-18, and IL-1β (serum levels *p* < 0.01, *p* < 0.01, *p* < 0.01, *p* < 0.05; mRNA expressions *p* < 0.05 vs. DOX for all). Administration of CAR alone did not significantly alter the serum levels and cardiac mRNA expressions of these inflammatory cytokines. Therefore, in addition to preventing DOX-induced oxidative stress, CAR may also prevent DOX-induced cardiotoxicity via downregulation of pathological inflammatory cytokines.

### CAR Inhibited Cardiomyocyte Apoptosis Induced by DOX

**FIGURE 5:**
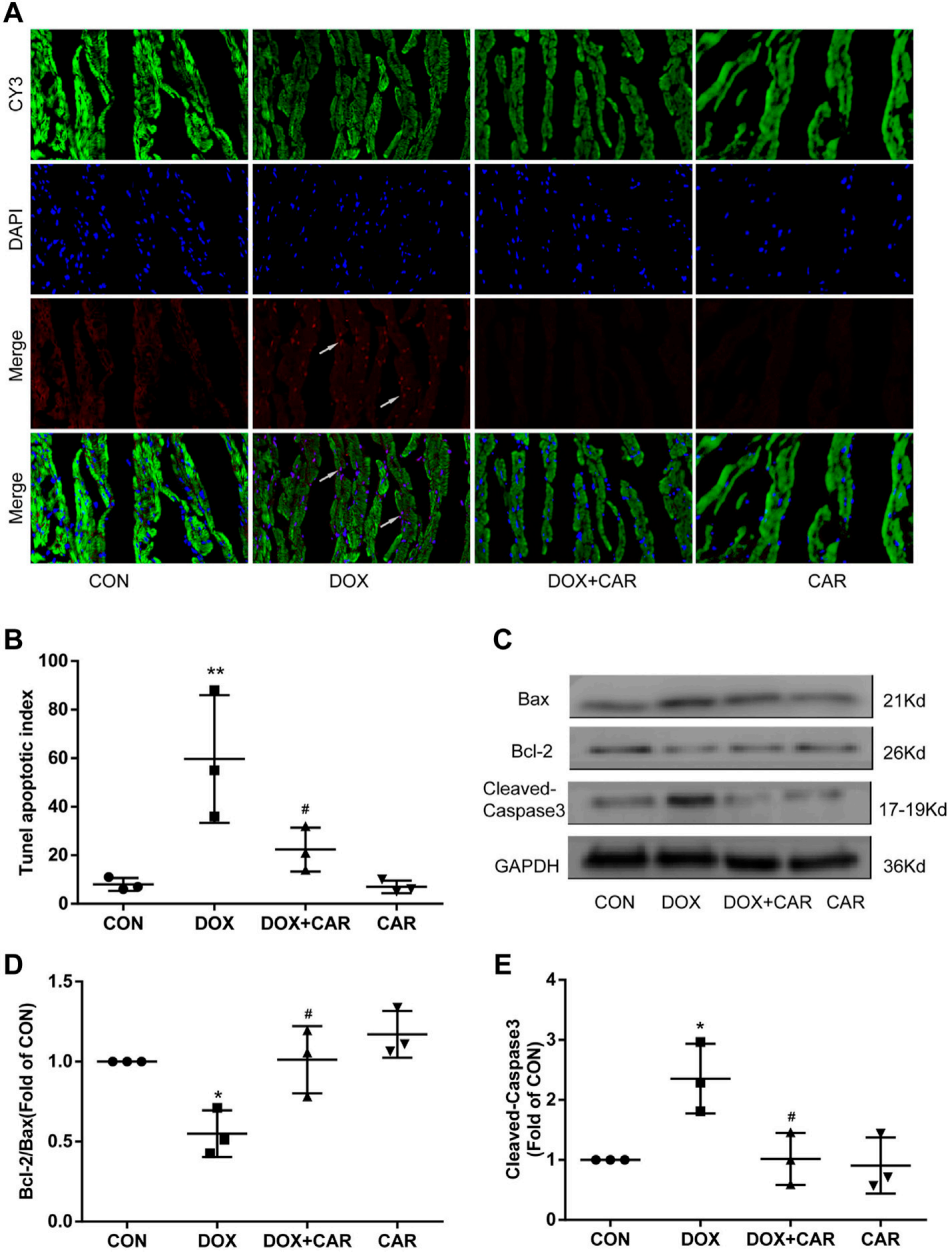
CAR inhibited cardiomyocyte apoptosis induced by DOX. **(A)**: Representative pictures of the TUNEL assay, **(B)**: Histogram representing quantitative analysis of the apoptotic index, **(C)**: Representative western blots of Bcl-2, Bax and Cleaved-Caspase-3, **(D)**: Histogram representing the quantitative analysis of the ratio of Bcl-2/Bax, **(E)**: Histogram representing the quantitative analysis of Cleaved-Caspase-3. Quantitative data are means ± SEM (n = 3). *p < 0.05, **p < 0.01 vs. CON; #*p* < 0.05 vs. DOX.

As DOX-induced cardiotoxicity is known to involve in the induction of apoptosis in cardiomyocytes, we tested whether the protective effect of CAR included the ability to prevent cardiomyocyte apoptosis by using TUNEL staining of heart tissues. As shown in Figure 5A, DOX caused a significant increase of apoptotic cells (pink staining) compared to CON group (*p* < 0.01 vs. CON, Figure 5B). CAR treatment was able to partially prevent DOX-induced increase of cardiomyocyte apoptosis (*p* < 0.05 vs. DOX), while CAR treatment alone had no significant effect.

To further investigate the ability of CAR to prevent DOX-induced apoptosis, we analyzed the apoptosis related proteins including Bcl-2, Bax and Cleaved-Caspase-3 in heart tissues using Western blot. As shown in Figures 5C, administration of DOX resulted insignificant increased expressions of Bax and Cleaved-Caspase-3, whereas the Bcl-2 protein level was significantly decreased. In contrast, CAR treatment markedly reversed DOX-mediated protein changes of Bcl-2, Bax and Cleaved-Caspase-3, as well as partially normalized the Bcl-2/Bax ratio (*p* < 0.05, *p* < 0.05 vs. DOX). No significant differences were detected in the protein levels of Bcl-2, Bax, and Cleaved-Caspase-3 between CAR and CON groups. It is tempting to speculate that this phenomenon may partially explain CAR’s ability to prevent DOX-induced cardiotoxicity by reducing apoptosis in cardiomyocytes.

### Effect of CAR on Akt/GSK-3β Signaling Following DOX-Induced Cardiotoxicity

**FIGURE 6:**
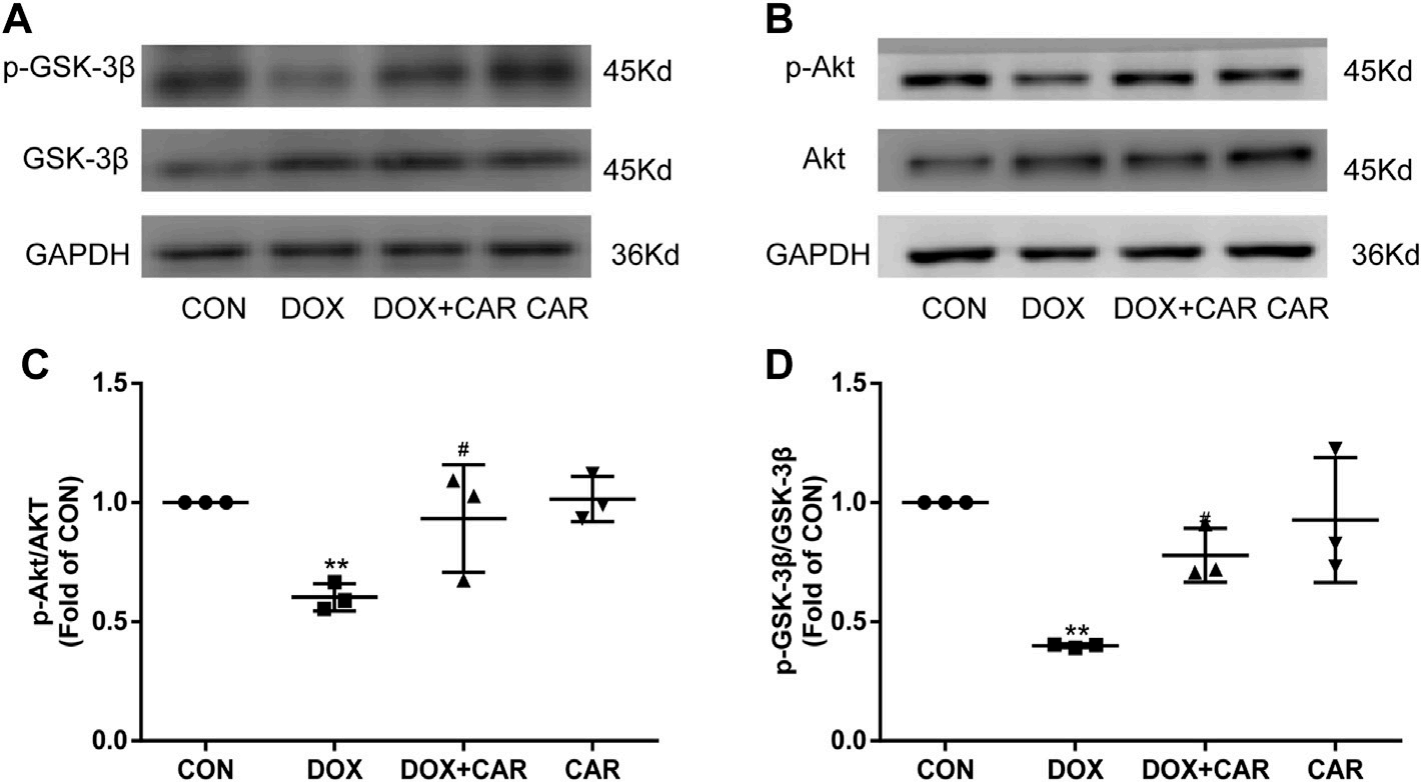
Effect of CAR on Akt/GSK-3β signaling following DOX-induced cardiotoxicity. **(A)**: Representative western blots of GSK-3β, **(B)**: Representative western blots of Akt, **(C)**: Histogram representing the quantitative analysis of GSK-3β, **(D)**: Histogram representing the quantitative analysis of Akt. Quantitative data are means ± SEM (n = 3). **p < 0.01 vs. CON; #*p* < 0.05 < 0.01 vs. DOX.

As previous studies have indicated that the cardioprotective and anti-hypertrophic effects of NHE1 inhibition were involved in the activation of Akt/GSK-3β signaling pathway (Javadov et al., 2009; Jung et al., 2010), we investigated the phosphorylation levels of Akt and its downstream target protein GSK-3β by Western blot. As shown in Figures 6A,B, the phosphorylation levels of Akt and GSK-3β significantly decreased by administration of DOX. In contrast, treatment with CAR attenuated DOX-induced decrease in phosphorylation of both Akt and GSK-3β (*p* < 0.05, *p* < 0.05 vs. DOX, Figures 6C,D). None of DOX or CAR significantly altered the total Akt and GSK-3β protein levels. These data suggest that CAR may protect against DOX-induced cardiotoxicity via activating Akt/GSK-3β survival signaling pathway.

### Sirt1 Involved in the Cardioprotective Effect of CAR Against DOX-Induced Cardiotoxicity

**FIGURE 7:**
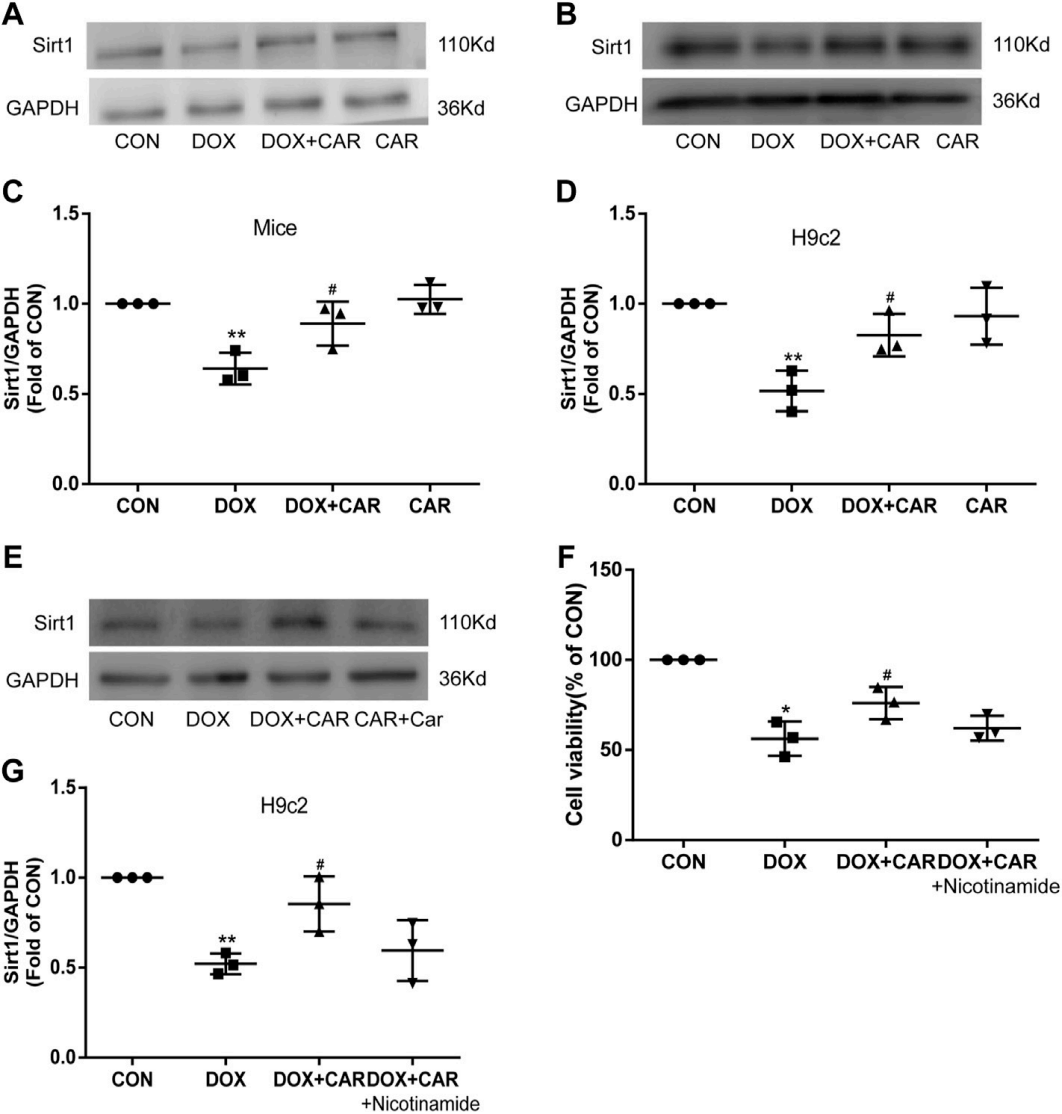
Sirt1 was involved in the cardioprotective effect of CAR against DOX-induced cardiotoxicity. **(A)**: Representative western blots of Sirt1 in cardiac tissues of mice, **(B)**: Representative western blots of Sirt1 in H9c2 cells, **(C)**: Histogram representing the quantitative analysis of Sirt1 in cardiac tissues of mice, **(D)**: Histogram representing the quantitative analysis of Sirt1 in H9c2 cells, **(E)**: Representative Western blots of Sirt1 in H9c2 cells treated with the inhibitor, **(F)**: The cell viability measured by CCK-8, **(G)**: Histogram representing the quantitative analysis of Sirt1 in H9c2 cells treated with inhibitor. Quantitative data are means ± SEM (n = 3). *p < 0.05, **p < 0.01 vs. CON; #*p* < 0.05 vs. DOX.

Previous studies have revealed that Sirt1 was decreased in DOX treated heart or H9c2 cardiac cells (Zhang S. et al., 2021; Liu Y. et al., 2021; Zhang WB. et al., 2021), and PI3K-Akt-GSK3β signaling pathway is required for Sirt1 induction by endoplasmic reticulum stress (Koga et al., 2015), we then tested the protein expression of Sirt1. Both in DOX treated mice and H9c2 cell, the protein expressions of Sirt1 decreased significantly, which were restored by CAR treatment (*p* < 0.05, *p* < 0.05 vs. DOX, Figures 7A–D). Nicotinamide, an inhibitor of Sirt1, reversed the protective effect of CAR by downregulating the protein expression of Sirt1 and reducing H9c2 cell viability (*p* < 0.05, vs. DOX, Figure 7E, G; *p* > 0.05 vs. DOX + CAR, and Figure 7F). These data suggest that activating Sirt1 pathway may mediate the cardioprotective role of CAR against DOX-induced cardiotoxicity.

## Discussion

Here, we demonstrated for the first time that CAR treatment suppressed DOX-induced cardiotoxicity, as indicated by improving cardiac function, reducing oxidative damage, inhibiting inflammatory response, and alleviating myocardial apoptosis. In addition, we found that Akt/GSK-3β signaling pathway was involved in the protective effects of CAR. Furthermore, the results from the present study also demonstrated that these protective effects of CAR were mediated by the activation of Sirt1 *in vivo* and *in vitro*, and Sirt1 inhibition abolished CAR treatment-mediated cardiac protection. Collectively, our data suggest that CAR may be a potential therapeutic drug and NHE1 may be a potential therapeutic target for the prevention and treatment of DOX-induced cardiotoxicity.

Doxorubicin is a well-established chemotherapeutic agent widely used in the treatment of hematological malignancies and solid tumors. Unfortunately, there are numerous serious toxicities associated with DOX treatment, particularly on the cardiovascular system, which severely limits its clinical use. The dose of DOX used in the present study (15 mg/kg) was comparable with the typical dose given to cancer patients (Venturini et al., 1996; Xiao et al., 2012). Our data demonstrated that this dosage of DOX resulted in cardiotoxicity in rats as evidenced by decreased body weight, elevated serum levels of cardiotoxicity biomarker enzymes, as well as alterations in heart function and cardiac histopathological damage. On the contrary, administration of CAR increased the body weight, attenuated the increased levels of serum CK-MB, LDH, and cTnT, improved the impairment of left ventricular function, as well as ameliorated the histopathological damage of the heart caused by DOX treatment, suggesting that CAR exerted a prominent protective effect against DOX-induced cardiotoxicity. In addition, the dose of CAR used in this experiment (10 mg kg^−1^ day^−1^) did not cause any cardiotoxicity, thus warranting further investigation into the possible molecular mechanisms underlying its cardioprotective effects.

Given the established role of oxidative stress in DOX-induced cardiotoxicity (Xu et al., 2001; Zhang et al., 2017), we first investigated the effect of CAR on biomarkers of oxidative stress in heart tissues. Excessive production of free radicals can cause oxidative damage to nearly all types of biological molecules, leading to numerous disease states (Halliwell and Gutteridge, 1990). Over-production of ROS leads to damage of nuclear and mitochondrial DNA, altered calcium homeostasis, decreased protein synthesis, and cardiomyocyte death. Numerous oxidative stress markers, including lipidperoxidation and lipid aldehydes, are found in heart tissues after DOX treatment (Chaiswing et al., 2004; Zhang et al., 2017). In agreement with this, our results showed that DOX-induced cardiotoxicity in rats was associated with significant increase of cardiac MDA level as well as reduced activities of the cardiac antioxidant enzymes (GSH-Px, SOD, and CAT). Notably, treatment with CAR significantly inhibited MDA production and prevented the decreased activities of cardiac GSH-Px, SOD, and CAT in rats subjected to DOX. A recent study indicated that DOX-induced ROS production in the H9c2 cells quantified with a fluorescent probe was inhibited by treatment with pinocembrin (Sangweni et al., 2020). In our results, H9c2 cells treated with DOX exhibited a clear increase in intracellular ROS level, which was reversed by CAR treatment. Therefore, suppressing oxidative stress may be one of the primary mechanisms by which CAR protects against DOX-induced cardiotoxicity.

In addition to directly causing deleterious effects, oxidative stress in the heart can also lead to inflammation, which is known to be involved in a variety of cardiovascular diseases, including atherosclerosis, atrial fibrillation, and inflammatory cardiomyopathies. Prior research has suggested that there is a strong association between cardiac oxidative stress and inflammatory cytokine release after DOX treatment (Bien et al., 2007). TNF-α, IL-6, IL-18, and IL-1β are the common proinflammatory cytokines involved in DOX-induced cardiotoxicity and are increased in individuals who have cardiac dysfunction (Miettinen et al., 2008; Tamariz and Hare, 2010). In agreement with the prior studies (Wang et al., 2016), we found that DOX application in rats led to increased serum levels and cardiac mRNA expressions of TNF-α, IL-6, IL-18, and IL-1β. CAR treatment prevented the increased serum levels of these proinflammatory cytokines and their cardiac mRNA expressions. It is interesting to speculate that alongside its antioxidant effects, anti-inflammatory properties may be also responsible for the protective effect of CAR against DOX-induced cardiotoxicity.

Beyond the damage inflicted by ROS and inflammation, myocardial apoptosis is believed to be the fundamental basis of DOX-induced cardiotoxicity (Kalay et al., 2006). β-Hydroxybutyrate, a small lipid-derived molecule derived from increased free fatty, could protect against DOX-induced cardiotoxicity by inhibiting cell apoptosis and oxidative stress and maintaining mitochondrial membrane integrity (Liu et al., 2020). Also, CAR treatment has been found to significantly suppress the induction of TUNEL-positive cardiomyocytes in cardiac hypoxia/reoxygenation. Therefore, we also examined the effect of CAR on myocardial apoptosis induced by DOX. As differential induction of apoptosis in cardiomyocytes caused by DOX treatment has been reported in different experimental animal models (Hou et al., 2006; Ruan et al., 2015; Argun et al., 2016), we observed increased TUNEL-positive cardiomyocytes following DOX treatment over a period of 12 days in rats, consistent with a previous study (Argun et al., 2016). Importantly, CAR treatment significantly inhibited DOX-induced myocardial cell apoptosis, thereby improving its myocardial toxicity.

In many cell types, apoptosis is regulated by Bcl-2 protein family. The pro-apoptotic Bax protein and anti-apoptotic Bcl-2 protein play a major role in determining whether or not cells undergo apoptosis. The translocation of Bax from the cytoplasm to mitochondria results in cytochrome c release from the mitochondria to promote apoptosis, while Bcl-2 can prevent the release of cytochrome c from mitochondria and suppress apoptosis progression (Scorrano and Korsmeyer, 2003). A recent study found that DOX administration increased Bax expression and decreased Bcl-2 expression in H9c2 cells (Liu et al., 2016). Prior research also suggested that CAR reduced mitochondrial Ca2+, the number of PI and TUNEL positive cells, cytosolic cytochrome c, caspase-3 activity, and ratio of Bax and Bcl-2 in primary cultured neonatal rat cardiomyocytes subjected to hypoxia/re-oxygenation (Sun et al., 2004). Our results revealed that treatment with DOX significantly reduced Bcl-2/Bax ratio and elevated the Cleaved-Caspase-3 protein expression in the heart, which was reversed significantly by administration of CAR. These results highly suggest that the cardioprotective effect of CAR is also due to its ability to regulate the levels of apoptosis related proteins. However, non-caspase-dependent apoptotic pathways can also be activated under DOX or cardiac IR condition, as evidenced by AIF translocation to the nuclei in H9c2 cardiomyoblasts treated with DOX (Moreira et al., 2014) and intracytosolic translocation of AIF and endonuclease G during cardiac ischemia (Yang et al., 2017). Therefore, whether AIF and mitochondrial endonuclease G are involved in the cardioprotective effect of Car against DOX-induced cardiotoxicity needs to be further studied.

Cell survival signaling pathways, including Akt, GSK-3β and ERK1/2, are known regulators of myocardial cell survival (Li et al., 2009), suggesting that they may be pharmacological targets for the prevention of myocardial cell apoptosis under stress conditions. Notably, it has previously been shown that DOX treatment caused a remarkable reduction of Akt and GSK3β phosphorylation in mouse heart (Prysyazhna et al., 2016), and erythropoietin protects against DOX-induced cardiotoxicity by activating PI3K-Akt-GSK-3β signaling pathway, thereby reducing cardiomyocyte apoptosis (Kim et al., 2008). Shenmai injection, one of the patented traditional Chinese medicine, prevents DOX-induced cardiotoxicity through activation of PI3K/Akt/GSK-3β signaling pathway (Li et al., 2020). In agreement with prior studies, we also observed that Akt and GSK-3β phosphorylation reduced in DOX-treated hearts. Treatment with CAR significantly attenuated DOX-induced decreased phosphorylated protein expressions of Akt and GSK-3β.

Sirt1 has been suggested to play key roles in redox regulation, cell apoptosis, and inflammation (Hwang et al., 2013). A previous study showed that exposure to DOX of H9c2 cells leads to cellular injury and the reduction of Sirt1 (Liu et al., 2016). Sirt1 is also highly expressed in cardiomyocytes and involved in DOX-induced cardiotoxicity (Dolinsky, 2017). Inconsistent with these findings, we also found that DOX reduced Sirt1 protein levels *in vivo* and *in vitro*. However, Zhang (Zhang et al., 2011) reported that Sirt1 level was slightly increased by DOX treatment, and resveratrol attenuated DOX-induced cardiomyocyte apoptosis in mice through up-regulation of Sirt1. The inconsistent expressions of SIRT1 under DOX condition may be related to the different animals used and the different action time of DOX, which may need further discussion. Of note, restoration of the expression of Sirt1 by CAR treatment could improve cardiac function and attenuate DOX-related cardiac injury in mice. Moreover, inhibition of Sirt1 by nicotinamide offset the protective effects provided by CAR treatment against DOX-induced H9c2 injury. These findings suggest that CAR exerts cardiac protection partially via activating Sirt1 signaling pathway. Sirt3, another NAD + -dependent histone deacetylase, restores mitochondrial respiratory chain defects, and cell viability of DOX treated cardiomyocytes (Blasco et al., 2018). Also, NHE-1 inhibitor EMD-87580 improves cardiac mitochondrial function through regulation of mitochondrial biogenesis during posti nfarction remodeling in these hearts (Javadov et al., 2006b), and attenuates the hypertrophic phenotype via improving mitochondrial integrity and decreasing generation of mitochondrial-derived reactive oxygen species (Javadov et al., 2006a). These evidences provide the relationship between DOX cardiotoxicity and Sirt3 or mitochondrial biogenesis. Further studies are needed to clarify the precise mechanism Sirt3 and mitochondrial biogenesis in the cardioprotective effect of CAR against DOX-induced cardiotoxicity.

## Conclusion

We demonstrate for the first time that CAR, a selective inhibitor of NHE1, exerts protective effects against DOX-induced cardiotoxicity via its antioxidant, anti-inflammatory, and anti-apoptotic activities. The results from the present study also demonstrate a role for Akt/GSK-3β and Sirt1 signaling pathway in the cardioprotective effects of CAR, and suggest that CAR may be a potential therapeutic drug and NHE1 may be a potential therapeutic target for the prevention and treatment of DOX-induced cardiotoxicity.

## Data Availability

The original contributions presented in the study are included in the article/supplementary material, further inquiries can be directed to the corresponding authors.
